# Data mining of mental health issues of non-bone marrow donor siblings

**DOI:** 10.1186/2043-9113-1-19

**Published:** 2011-07-20

**Authors:** Morihito Takita, Yuji Tanaka, Yuko Kodama, Naoko Murashige, Nobuyo Hatanaka, Yukiko Kishi, Tomoko Matsumura, Yukio Ohsawa, Masahiro Kami

**Affiliations:** 1 Division of Social Communication System for Advanced Clinical Research, the Institute of Medical Science, the University of Tokyo, 4-6-1 Shirokanedai, Minato-ku, Tokyo 108-8639, Japan; 2 Department of Systems Innovation, School of Engineering, the University of Tokyo, 7-3-1 Hongo, Bunkyo-ku, Tokyo 113-8656, Japan

**Keywords:** hematology, transplantation, data mining, Scenario Map analysis, physician-patient communication

## Abstract

**Background:**

Allogenic hematopoietic stem cell transplantation is a curative treatment for patients with advanced hematologic malignancies. However, the long-term mental health issues of siblings who were not selected as donors (non-donor siblings, NDS) in the transplantation have not been well assessed. Data mining is useful in discovering new findings from a large, multidisciplinary data set and the Scenario Map analysis is a novel approach which allows extracting keywords linking different conditions/events from text data of interviews even when the keywords appeared infrequently. The aim of this study is to assess mental health issues on NDSs and to find helpful keywords for the clinical follow-up using a Scenario Map analysis.

**Findings:**

A 47-year-old woman whose younger sister had undergone allogenic hematopoietic stem cell transplantation 20 years earlier was interviewed as a NDS. The text data from the interview transcriptions was analyzed using Scenario Mapping. Four clusters of words and six keywords were identified. Upon review of the word clusters and keywords, both the subject and researchers noticed that the subject has had mental health issues since the disease onset to date with being a NDS. The issues have been alleviated by her family.

**Conclusions:**

This single subject study suggested the advantages of data mining in clinical follow-up for mental health issues of patients and/or their families.

## Introduction

Allogeneic hematopoietic stem cell transplantation (allo-HSCT) has been established as a treatment for hematologic malignancies such as leukemia and malignant lymphoma and is the only way to cure patients with advanced stage hematologic malignancies [1,2]. In Japan, allo-HSCTs were conducted on 2,242 cases in 2008 with a total of 33% of donors for the allo-HSCTs being siblings or relatives [3]. Several reports demonstrated that donating bone marrow or hematopoietic stem cells in peripheral blood can affect the donor's safety and quality of life, thus the donor's safety and quality of life should be carefully considered during allo-HSCT [4,5].

Undergoing allo-HSCT also increases the likelihood of patients and their families developing mental health issues [6-10]. Donor selection from relatives can occasionally cause psychological conflicts between a donor and other relatives, including non-donor siblings (NDS), which would result in difficult management for continuous medical follow-up. This is a practical concern but has not been well studied in previous reports [11,12].

Data mining allows processing a large, multidisciplinary data set. Its effective applications into medical fields are highly desired since health care information has been dramatically increased and diversified [13,14]. Currently, the data mining approach has been applied to several clinical and biomedical fields (Table 1). For example, a data detection system has been proposed in the development of electronic health records to discover new findings, leading to efficient and safe clinical practice [15,16]. In the genomics and proteomics field, data mining contribute their analysis as multidimensional tests, cluster analysis and pathway analysis [17-19]. The concept of data mining algorithm can be divided into two groups in the medical field; supervised and unsupervised approach [20]. The supervised approach is a traditional style of data analysis where prepared hypotheses are tested to evaluate the statistical significance, accuracy and validity. The unsupervised approach is a process to explore new knowledge called 'knowledge discovery'. Knowledge discovery is an excellent tool to generate new hypotheses effectively as shown by some reports with a text mining method on literature review and medical records [21-24]. Herein we thought that knowledge discovery would provide us unanticipated and useful keywords or relationships from clinical interviews, leading to better clinical follow-up.

**Table 1:** Conceptual differences of data mining approach.

Research area	Electronic medical record	Genomics/Proteomics	This study: Mental health on NDS
Data source	Physicians/nurses' Description, laboratory data and radiologic images on medical record	Gene expression data from cDNA microarray/mass spectrometry	Interview with the subject

Expected results	Automatic and effective data extraction/sorting	Extraction of genes/proteins with statistical significance Classification of gene/proteins Visualization of gene/protein expression pattern or pathway	Extraction of important and rarely-appeared words Visualization of relationship between keywords

Concept*	Supervised/Unsupervised approach	Supervised/Unsupervised approach	Unsupervised approach

Representative algorism of data mining technique	Data extraction matching with prepared data criteria	To provide statistically meaningful analysis for high-throughput and multi-dimensional biological data in the association with phenotype	To discover unanticipated, rarely appeared key-elements by Scenario Map analysis

Aims	Linking between medical record description and research issues To develop effective and commonly available electronic health record	To discover new biomarker or diagnostic method To discover therapeutic target	For better clinical follow-up by understanding unanticipated individual concerns

Conceptual differences of data mining approach in representative medical research areas are shown. *Supervised approach aims for testing or validation of hypothesis while unsupervised approach used for discovering unanticipated events or knowledge.

**Figure 1:**
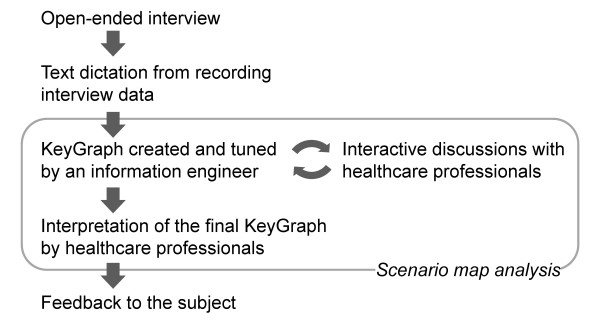
**A working flow**. The subject was interviewed using open-ended question style and text data of the interview was generated. KeyGraph was created and tuned by an information engineer in discussion with healthcare professionals. The final KeyGraph was interpreted in detail by healthcare professionals and provided the subject the feedback. Scenario Map analysis includes interactive framework between computer outputs by an information engineer and healthcare professionals to obtain a comprehensive graph.

The Scenario Map analysis is a new approach of knowledge discovery where the relationships among keywords in plain texts can be visualized as a diagram called KeyGraph [25,26]. The Scenario Map allows figuring out important keywords linking different conditions/events even though they are infrequently using words, and in turn discovering new findings or knowledge through the human-computer interaction process. This process is the repeated circle between computer outputs of KeyGraph from dataset and the interpretation by humans (Figure 1). Successful studies with the Scenario maps in clinical laboratory tests and designing new products have already been reported [27,28]. Thus the extended study using this novel data mining approach to mental health care for NDS should be considered although few reports with the approach have been demonstrated to date. This is the first report focusing on the mental health issues of a NDS using the Scenario map.

## Case description

### Case summary

The subject is a 47-year-old woman. When her younger sister developed chronic myeloid leukemia, she was 27 years old and living in the United States with her husband and their two children, apart from her parents and her younger sister since her marriage. The subject shared information on the treatment of leukemia with her sister at the disease onset and learned about allo-HSCT for the first time. She had a positive sense of allo-HSCT; however she did not match with her younger sister for human leucocyte antigen (HLA). Thus, she was not selected as a donor and the bone marrow transplantation was performed with her mother as the donor. Twenty years have passed since the transplantation and the subject's younger sister was still living at the time of this study.

The subject was interviewed by a hematologist who was not involved in the transplantation. The open-ended interview was carried out without prepared questions to avoid misleading results by interviewers. The subject voluntarily talked about the clinical course in her younger sister from the disease onset until the present day including her sense, feelings, family-relationships and job. The subject participated in this study voluntarily and consented to the interview being recorded and analyzed by an information engineer.

This study was approved by the Institutional Review Board of The Institute of Medical Science, The University of Tokyo (19-19-1105).

### Scenario Map analysis

**Figure 2:**
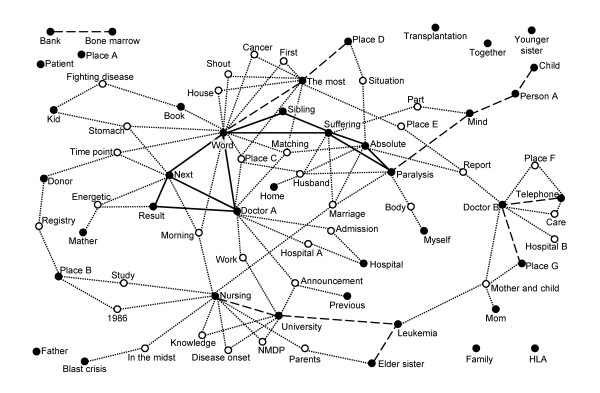
**Key Graph**. Black and white nodes indicate high and less frequently used words in the interview, respectively. The solid, dashed and dotted line indicates degree of co-occurrence between nodes as high, middle and low level, respectively. White nodes indicate words that appeared less frequently in the interview. Personal information was exchanged to general words before submission of the manuscript. Abbreviations; NMDP: the National Marrow Donor Program, HLA: Human Leukocyte Antigen.

The recorded data was dictated to use as plain text data. The independent information engineer created a KeyGraph as previously described [25,26]. First, word frequency and the co-occurrence of words, meaning the coefficients on paired words in the same sentence, were determined (Table 2). Then, a well-experienced information engineer programmed settings on highly-frequent and tightly-paired words repeatedly to obtain a comprehensive KeyGraph in discussion with physicians and a nurse, since the definition of high frequency and co-occurrence can influence keyword clustering [26]. This human-computer interaction is an important step in Scenario Map Analysis allowing creative ideas in investigators. In this study, highly-frequent words were defined as words that appeared more than 6 times in the interview. The KeyGraph can visualize relationship among main structure as cluster consisted of highly-frequent and co-occurrent words (block nodes and solid lines in Figure 2) and words that appeared infrequently (white nodes). The white nodes linking between main structures are keywords, which should be focused on in this analysis.

**Table 2:** The list of words in frequency and co-occurrence order.

Cluster	Word	Frequency
Pre-transplant	Sibling	10

	The most	9

	Next	8

	Place D*	8

	Doctor A*	7

	Word	6

	Results	6

		

Emotion	Child	126

	Mind	15

	Person A*	11

	Suffering	10

	Paralysis	7

	Absolute	6

		

Transplantation process	Place G*	12

	Telephone	10

	Doctor B*	7

		

Subject's life	Elder sister	16

	Leukemia	9

	Nursing	8

	University	7

		

Other**	Younger sister	50

	Myself	48

	Bone marrow	46

	Father	44

	Transplant	43

	Mother	42

	Previous	24

	Patient	23

	Kid	21

	Place A*	21

	Bank	18

	Place B*	16

	Donor	15

	Hospital	15

	Blastic crisis	14

	Mom	12

	Family	10

	HLA	10

	Home	10

	Together	7

	Book	6

Words appearing more than 6 times in the interview were defined as high-frequency in this study. Words in the same cluster have high co-occurrence each other. *Replaced words to protect personal information. **Words independently placed or had low-levels of co-occurrence with the other words in KeyGraph.

Medical doctors and a nurse discussed relationships among clusters and keywords in the final KeyGraph and generated hypotheses on her mental health issue. The KeyGraph and hypotheses were sent via e-mail to the subject in order to validate them. Figure 1 shows a working flow of this study.

### Interpretation of KeyGraph

**Figure 3:**
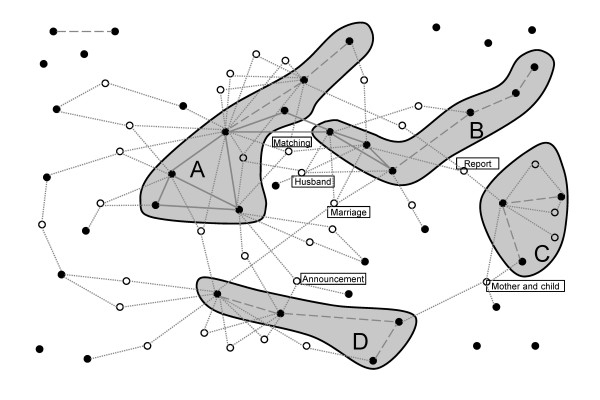
**Interpretation of KeyGraph**. The clusters and the keywords were extracted based on the interpretation of Figure 2. Each cluster was named by pre-transplant (A), emotion (B), transplant process (C) and subject's life (D). Keywords were shown as boxed text.

A total of one hour and 11 minutes was taken for the interview. Based on the discussion among physicians and a nurse using KeyGraph, the following four clusters were indentified: pre-transplant, emotion, transplant process, and subject's life (Figure 3). Furthermore, we extracted 'mother and child', 'announcement', 'report', 'matching', 'marriage', and 'husband' as keywords linking the clusters (Figure 3).

The emotion cluster includes frequently used words of 'suffering', 'absolute', 'paralysis', 'mind', 'Person A' and 'child'. Among them, the word 'paralysis' was used as a 'paralysis of the mind' to express a condition where the subject was unable to control her emotions because of mental stress. In addition, Person A was a younger child of NDS similar to the subject and the subject projected her feeling onto Person A in the interview. A high-frequency word of 'myself' is linked with the emotion cluster via 'body'. These findings deduced that the subject suffered emotional distress related to the treatment of her younger sister.

'Marriage', 'husband' and 'mother and child' are keywords linking clusters, suggesting that they would play an important role for the subject. Especially, 'marriage' is a keyword linking between emotion and subject's life clusters. The subject was already married when her sister developed symptoms of leukemia. In contrast, the words 'father', 'family' and 'younger sister', which should be closely related to the subject herself, were not linked with any words and clusters in the KeyGraph. Twenty years ago, it was difficult to conduct bone marrow transplantation without sibling donors since there was no bone marrow bank in Japan at that time. In this case, the subject was a NDS because of HLA mismatch. Considering these backgrounds and links in the KeyGraph together, the analysts interpreted that the subject had a feeling of isolation from her family due to being a NDS and that the subject was mentally supported by her husband or mother. Of note, the links between emotion cluster, 'husband' and 'marriage' might suggest negative impact on her mind since emotion cluster represents psychological suffering.

'Report' is a keyword that connected with the transplant process and emotions cluster. Similarly, 'announcement' is linking between pre-transplant and subject's life cluster. According to our discussions, the emotional distress was related to 'report' on her sister's treatment such as the results of laboratory tests and clinical examinations and announcement of disease would have an influence on the subject's life before transplantation.

Based on the interpretations described above, we hypothesized that the subject suffered from emotional distress related to her sister's treatment and that husband and mother was a psychological mainstay for her.

The two figures were presented to the subject while our interpretations and hypothesis were not shown to her in order to avoid misleading conclusions. After reviewing the KeyGraphs, the subject said that she has had psychological stress because of the fact that she was not selected as the donor during the subsequent course of her sister's treatment and that currently she had mental health issues of being a NDS. Furthermore, when she saw the keywords 'husband' and 'married', which were linked to the emotion cluster with the others, she realized that her husband kindly supported her. This was consistent with our hypothesis obtained from discussions using the Scenario Map analysis.

## Discussion

This is the first report to implement the Scenario Map analysis as a novel data mining tool into the qualitative assessment of mental health on NDSs although preliminary conclusions with caution should be regarded on this paper due to the nature of single case study. Psychological issues among patient families can be developed with bone marrow transplantation [29-31]. However, the long-term, psychological impact of the transplantation on NDS has not been well studied to date [11,12]. Of note, the subject in this study has had emotional distress for more than 20 years since the transplant, suggested by the interpretation of KeyGraph. This might be related to her feelings of alienation due to not being a donor. The assessment of mental health issues on NDSs using Scenario Map analysis should be studied with a large cohort and we are planning further studies with similar cases.

In this study, Scenario Map analysis was used for a data mining tool and enabled both clinicians and the subject to be aware of the new findings on mental health issues for NDS. It was also helpful to notice that the NDS's psychological stress can be healed by family's support through the process of the Scenario Map. Since the subject has known that she felt a psychological stress related to her younger sister's treatment, the words indicating emotional conditions appeared frequently in the interview. On the contrary, she did not mention her family's support in the interview, but recognized it after reviewing the KeyGraph. Regarding stress coping, self-recognition of familial support is beneficial to reduce her/his anxiety [32]. Medical interview with the Scenario map would improve clinical management of bone marrow transplant patients and their families including psychological problems.

Clinical relevance of the findings presented here would be helpful for patient/family support during or after allo-HSCT rather than donor selection since donor selection from family is usually performed on the basis of biological assessment of HLA matching and physical tolerability for hematopoietic stem cell harvest [33,34]. Previous paper showed that better scores on family support were associated with decreased risk of mortality or reduced patients' anxiety, suggesting that psycho-social care for patient family should be considered for better treatment outcome [29,35,36]. Therefore the approach in this case presentation suggests clinical availability in psycho-social care.

A major research method on psycho-social care for patient family is interview-based, qualitative approach and fewer quantative studies [12]. This might be explained by the difficulty to point out key issues from individual experiences of different patient/family. Text data mining is beneficial in such circumstance since data mining allows both aspects of research style; quantative approach such as frequency and co-occurrence of words and qualitative study like interpretation of the interview. This manuscript also showed a new field to bridge between mental health care and text data mining, suggesting novel collaborations between clinicians and information engineers.

There are some limitations in this approach; KeyGraph has flexibility to allow creative hypothesis generation but reproducibility of the graph is limited since the settings of high frequency and co-occurrence depend on analysts' perceptions to obtain a comprehensive graph. Therefore Scenario Map analysis should be used for discovering new hypotheses, not for validation study. Also analysts should know the background of the objectives to interpret KeyGraph effectively as analysts understood social background of all-HSCT in this study. The combination of Scenario Map analysis and subsequent traditional style of statistical study would be a more powerful tool to create new findings with liability and this study positions at the initial stage of the series.

## Conclusions

This case study suggests the following points: NDSs may have a long-term emotional distress, family support is important in solving it, and the Scenario Map analysis can be useful to assess NDS's mental health issues. Thus, this case report proposed an informative method in mental health care after bone marrow transplantation although this report shows preliminary results with single case indicating limited usefulness and reliability. The methodology in this study needs to be validated in an extensive study with a large number of cases.

## Abbreviations

Allo-HSCT: allogenic hematopoietic stem cell transplantation; NDS: non-donor siblings; HLA: human leukocyte antigen.

## Competing interests

The authors declare that they have no competing interests.

## Authors' contributions

MT participated in the study design, interpretation of results, discussion and preparation of the manuscript. YT participated in the study design, coordination, interview, interpretation of results and discussion, and helped to prepare the manuscript. YKO participated in the study design, coordination, interpretation of results and discussion. NM participated in study design and discussion. NH participated in coordination and discussion. YKI participated in study design and discussion, and helped to draft the manuscript. TM participated in coordination and discussion. YO participated in information engineering and discussion. MK participated in the study design, discussion and preparation of the manuscript. All authors read and approved the final manuscript.
